# Family-specific, novel, deleterious germline variants provide a rich resource to identify genetic predispositions for *BRCAx* familial breast cancer

**DOI:** 10.1186/1471-2407-14-470

**Published:** 2014-06-26

**Authors:** Hongxiu Wen, Yeong C Kim, Carrie Snyder, Fengxia Xiao, Elizabeth A Fleissner, Dina Becirovic, Jiangtao Luo, Bradley Downs, Simon Sherman, Kenneth H Cowan, Henry T Lynch, San Ming Wang

**Affiliations:** 1 Department of Genetics, Cell Biology and Anatomy, College of Medicine, University of Nebraska Medical Center, 986805 Nebraska Medical Center, Omaha, NE 68198, USA; 2 Hereditary Cancer Center, Department of Preventive Medicine, Creighton University School of Medicine, 2500 California Plaza, Omaha, NE 68178, USA; 3 Fred & Pamela Buffett Cancer Center, Omaha, USA; 4 Department of Biostatistics, College of Public Health, University of Nebraska Medical Center, Omaha, USA; 5 Department of Medicine, Creighton University School of Medicine, 2500 California Plaza, Omaha, NE 68178, USA

## Abstract

**Background:**

Genetic predisposition is the primary risk factor for familial breast cancer. For the majority of familial breast cancer, however, the genetic predispositions remain unknown. All newly identified predispositions occur rarely in disease population, and the unknown genetic predispositions are estimated to reach up to total thousands. Family unit is the basic structure of genetics. Because it is an autosomal dominant disease, individuals with a history of familial breast cancer must carry the same genetic predisposition across generations. Therefore, focusing on the cases in lineages of familial breast cancer, rather than pooled cases in disease population, is expected to provide high probability to identify the genetic predisposition for each family.

**Methods:**

In this study, we tested genetic predispositions by analyzing the family-specific variants in familial breast cancer. Using exome sequencing, we analyzed three families and 22 probands with *BRCAx (BRCA-*negative) familial breast cancer.

**Results:**

We observed the presence of family-specific, novel, deleterious germline variants in each family. Of the germline variants identified, many were shared between the disease-affected family members of the same family but not found in different families, which have their own specific variants. Certain variants are putative deleterious genetic predispositions damaging functionally important genes involved in DNA replication and damaging repair, tumor suppression, signal transduction, and phosphorylation.

**Conclusions:**

Our study demonstrates that the predispositions for many *BRCAx* familial breast cancer families can lie in each disease family. The application of a family-focused approach has the potential to detect many new predispositions.

## Background

Breast cancer is a leading cancer in women [1]. About 10-20% of breast cancer cases are family clustered, with multiple family members affected by the disease [2]. Genetic predispositions are the major risk factor for the disease. However, the genetic predispositions are currently known for only 30-40% of the familial breast cancer disease families. The remaining 60-70% of women with familial breast cancer have unknown predispositions and are diagnosed with *BRCAx,* for their unknown predisposition of familial breast cancer [3]. It is estimated the “*missing”* heredity trait for *BRCAx* families likely consists of thousands of rare variants, each presenting a minor disease risk [4]. Indeed, broadly screening the variants across disease populations has uncovered multiple new genetic predispositions for familial breast cancer. A consistent pattern among these newly classified predispositions is that they are always present at very-low frequencies in the given disease population [5-10]. Their extreme rarity implies that a greater sampling size of disease populations is required to identify the germline predispositions [10]. However, such an expansion is deemed to increase the complexity of data analysis, experimental costs, and time needed. As such, focusing only on the rare variants will not likely be able to determine the entire spectrum of genetic predispositions for *BRCAx* familial breast cancer families. New alternative hypotheses and approaches must be explored to improve the situation. For example, mosaic mutation has implications as potential predispositions for familial breast cancer [11].

Familial breast cancer is defined as an autosomal dominant genetic disease [12]. Although incidences of breast cancer often exhibit atypical Mendelian patterns due to the factors such as low penetrance of genetic predispositions, the predisposition in a disease-prone family is expected to transmit across generations and shared between family members. Focusing on each disease family with a history of the disease is expected to improve the chance to detect the predisposition in a family compared to screening the disease population of pooled cases without family relationships, which can dilute the predisposition highly prevalent in a disease family into insignificant level.

We hypothesize that the unknown predispositions for many *BRCAx* familial breast cancer are specific to each family with a history of the disease. Our previous exome study of a *BRCAx* familial breast cancer family shows the presence of rich genetic variants [13]. In the present study, we expand the exome sequencing study by analyzing three families with *BRCAx* familial breast cancer; 17 members had cancer, and five members were without cancer. Our study also includes 22 probands of *BRCAx* familial breast cancer. Our study reveals the presence of family-specific, novel, deleterious genetic variants as putative genetic predispositions in each family with *BRCAx* familial breast cancer.

## Methods

### Use of human subjects

The use of the patient samples for the study was approved by the Institutional Review Boards (IRB) of Creighton University School of Medicine (#00-12265 ) and University of Nebraska Medical Center (718-11-EP). All subjects signed the Consent to Participate Form for cancer genetic study.

**Figure 1:**
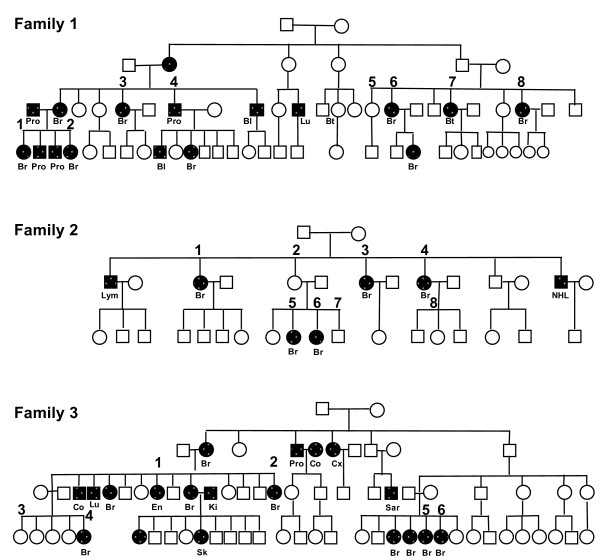
**Pedigrees of the three families used in the study.** BC (breast cancer), Bt (brain tumor), CRC (colorectal cancer), Lu (lung cancer), En (endometrium cancer), Ki (kidney cancer), Lym (lymphoma), NHL (non-Hodgkin lymphoma), OC (ovarian cancer), Pro (prostate cancer). Sar (sarcoma), Sk (skin cancer).

Individuals from three families with *BRCAx* breast cancer were used to generate exome sequences as we have previously described [13]. *Family I* included six individuals with breast cancer and two individuals without breast cancer. *Family II* included five individuals with breast cancer, one obligate carrier and two individuals without breast cancer. *Family III* included five individuals with breast cancer and one individual without breast cancer. Additionally, 22 probands for *BRCAx* familial breast cancer were included in exome sequencing. All cases used in the study were *BRCA1*-negative, and *BRCA2*-negative, 41 were female and 3 were male, the average age is 42 years old (Figure 1, Table 1).

**Table 1:** BRCAx familial breast cancer cases used in the study

**Family**	**Cancer type**	**Pathology**	**BRCA1/2**	**Exome**				
				**Reads**	**Bases**	**Bases map rate (%)**	**Coverage**	**Variant called**
Family 1								
1	Breast	Infiltrating ductal	**-**	42,973,730	4,340,346,730	97.6	70	184,865
2	Breast	Not available	*-*	40,158,059	4,055,963,959	98.3	65	152,692
3	Breast	Infiltrating ductal	**-**	46,240,754	4,670,316,154	97.2	75	176,554
4	Prostate	Adenocarcinoma	**-**	23,418,595	2,365,278,095	98.1	38	207,103
5	No Cancer		**-**	40,313,161	4,071,629,261	98.0	66	213,347
6	Breast, Colon	Adenocarcinoma	**-**	17,496,012	1,767,097,212	97.9	28	183,741
7	Brain	Not available	**-**	36,166,319	3,652,798,219	99.5	59	171,425
8	Breast	Adenocarcinoma	**-**	27,830,687	2,810,899,387	96.3	45	104,343
Family 2								
1	Breast, Breast	Medullary, infiltrating ductal	**-**	33,419,098	3,375,328,898	92.9	54	113,079
2	Obligated carrier		**-**	27,261,117	2,753,372,817	92.4	44	115,328
3	Breast	Infiltrating ductal	**-**	40,973,473	4,138,320,773	99.6	67	127,272
4	Breast	Ductal carcinoma in situ	**-**	29,561,523	2,985,713,823	91.5	48	108,655
5	Breast	Infiltrating ductal	**-**	25,790,969	2,604,887,869	93.1	42	84,687
6	Breast	Infiltrating ductal	**-**	37,657,589	3,803,416,489	91.6	61	139,891
7	No Cancer		**-**	17,433,912	1,760,825,112	91.6	28	131,786
8	No Cancer		**-**	35,977,512	3,633,728,712	97.3	59	128,680
Family 3								
1	Endometrial	Adenocarcinoma	**-**	33,662,978	3,399,960,778	93.2	55	129,754
2	Breast, Skin	Basal, infiltrating ductal	**-**	29,648,460	2,994,494,460	98.3	48	198,862
3	No Cancer		**-**	53,411,156	5,394,526,756	98.8	87	193,017
4	Breast	Infiltrating ductal	**-**	31,736,845	3,205,421,345	98.3	52	130,941
5	Breast	Ductal carcinoma in situ	**-**	35,014,538	3,536,468,338	98.4	57	129,754
6	Breast	Not available	**-**	38,418,769	3,880,295,669	97.5	62	161,953
Probands								
1	Breast	Ductal carcinoma in situ	**-**	17,832,681	1,801,100,781	93.1	29	109,864
2	Breast	Invasive ductal carcinoma	**-**	36,166,319	3,652,798,219	99.5	59	142,155
3	Breast	Invasive ductal carcinoma	**-**	50,944,516	5,145,396,116	98.4	83	152,125
4	Breast	Invasive ductal carcinoma	**-**	43,889,986	4,432,888,586	99.6	71	169,633
5	Breast	Invasive ductal carcinoma	**-**	40,125,408	4,052,666,208	99.5	65	153,511
6	Breast	Invasive lobular carcinoma	**-**	31,798,628	3,211,661,428	97.5	52	119,875
7	Breast	Invasive ductal carcinoma	**-**	49,739,415	5,023,680,915	99.6	81	113,058
8	Breast	Invasive ductal carcinoma	**-**	63,352,269	6,398,579,169	99.6	103	99,732
9	Breast	Invasive ductal carcinoma	**-**	43,744,840	4,418,228,840	99.5	71	149,873
10	Breast	Invasive ductal carcinoma	**-**	43,573,311	4,400,904,411	99.6	71	141,236
11	Breast	Invasive ductal carcinoma	**-**	40,938,838	4,134,822,638	99.3	67	143,262
12	Breast	Ductal carcinoma in situ	**-**	36,258,870	3,662,145,870	99.6	59	138,018
13	Breast	Ductal carcinoma in situ	**-**	34,550,745	3,489,625,245	99.4	56	146,858
14	Breast	Invasive ductal carcinoma	**-**	50,295,200	5,079,815,200	99.5	82	156,666
15	Breast	Invasive ductal carcinoma	**-**	60,736,566	6,134,393,166	99.7	99	115,909
16	Breast	Invasive ductal carcinoma	**-**	57,383,360	5,795,719,360	99.6	93	120,945
17	Breast	Invasive ductal carcinoma	**-**	44,922,611	4,537,183,711	99.6	73	110,503
18	Breast	Invasive ductal carcinoma	**-**	33,883,509	3,422,234,409	99.4	55	131,955
19	Breast	Invasive ductal carcinoma	**-**	49,729,619	5,022,691,519	99.5	81	146,665
20	Breast	Invasive ductal carcinoma	**-**	63,184,143	6,381,598,443	99.6	103	119,680
21	Breast	Invasive ductal carcinoma	**-**	28,002,381	2,828,240,481	99.6	46	86,924
22	Breast	Invasive ductal carcinoma	**-**	47,794,798	4,827,274,598	99.5	78	112,030
Average	38,941,211	3,933,062,277	97.7	63	140,187			

### Exome sequencing

For each sample, exome sequencing used DNA from blood cells. Exome libraries were constructed using the TruSeq Exome Enrichment Kit (62 Mb, Illumina, San Diego, CA) as per manufacturer’s procedures. Exome sequences were collected with a HiSeq™ 2000 sequencer (Illumina, San Diego, CA) with paired-end (2 × 100). All exome data were deposited in the Sequence Read Archive (SRA) database in the National Center for Biotechnology Information (NCBI) (Accession numbers SAMN02404413- SAMN02404456).

### Exome sequence mapping and variant calling

Exome sequences were mapped to the human genome reference sequence hg19 by Bowtie2 with default parameters in paired mode [14]. The subsequent SAM files were converted to BAM files. Duplicates were removed using Picard (http://picard.sourceforge.net). The mapped reads were locally realigned using the genome mapping tool RealignerTargetCreator from the Genome Atlas Tool Kit (GATK) [15]. The base quality scores were recalibrated using BaseRecalibrator (GATK), with NCBI dbSNP build 137, in the GATK resource bundles for reference sequence hg19. VarScan 2 was used for variant calling, [16]. VarScan 2 was run on pileup data generated from BAM files using SAMtools utilities [17]. The mpileup command, with –B parameter to disable base alignment quality (BAQ) computation, and the default parameters were used, with the minimum read depth at 10 and the minimum base quality at 30. The called variants were annotated with ANNOVAR using the software-provided databases of the Reference Sequence (RefSeq; NCBI), dbSNP 137, the 1000 Genomes Project, and the NIH Heart, Lung and Blood Institute (NHLBI) Exome Sequencing Project (ESP) 6500 (http://evs.gs.washington.edu).

Those that matched in the databases were classified as known variants and removed. Family-specific normal variants were eliminated by removing the variants shared between the affected and the unaffected family members in each family. The remaining novel variants were classified into synonymous, non-synonymous, splicing site change, stop gain- or loss groups. The variants causing synonymous changes were then removed. For the remaining variants, PolyPhen-2 was used to identify variants causing deleterious effects in the affected genes [probably damaging score: 0.909-1; possibly damaging score: 0.447 - 0.908; Benign score: 0 - 0.446; HumVar score: [18]. The variants defined as benign were removed. These processes generated a list of novel, deleterious variants only present in the cancer-affected family members and probands, Note that the variants in probands were filtered by population databases only.

### Power calculation

Using a two-sided paired *t*-test and assuming a genetic relative risk (GRR) equal to 5.8, disease prevalence equal to 0.03, a disease locus frequency equal to 0.01, and a sib recurrence ratio of 2, a sample size of 20 achieves 81% power to detect a mutation difference with a (standardized) effect size of 0.67 between the affected member and the unaffected member. The significance level (alpha) is, in turn, 0.05 [19,20].

### Validation

Sanger sequencing was used to validate deleterious variants. Sense and antisense PCR primers for each selected variant were designed using the Primer3 program. The original DNA samples that were used in exome sequencing were served as PCR templates. PCR amplicons were subjected to BigDye sequencing. The resulting sequences were evaluated using CLC Genomics Workbench Program (Cambridge, MA) to confirm the variants called from exome sequences.

## Results

### Mapping exome data and calling variants

Exome sequences were collected via a blood sample from each study participant and mapped to the human genome reference sequence hg19. Variants were called from the mapping data. We focused on single-base, non-synonymous variants that affect protein coding, splicing, and stop gain- or loss mutations, which are reliably detectable by exome analysis [21]. The average exome coverage was 63x, and the average number of variants called was 140,187 per case (Table 1).

To increase the likelihood that the variants identified in the breast cancer-affected family members are breast cancer-associated, variants in each data set were filtered by: 1) removal of common variants present in human populations. All variants matching to population-derived variant databases (i.e., dbSNP137, ESP6500, and 1000 genomes) were removed; 2) Removal of family-specific normal variants. For the three families in the study, the variants shared between the affected and the unaffected members in the same family were removed. To identify those causing deleterious effects in the affected genes, the remaining variants were analyzed using the Polyphen-2 Program [18]. A total of 337 novel, deleterious variants present only in the affected members of Families I, II, and III were identified at an average of 112 variants per family (Table 2, Additional files 1: Table S1A, B, C); 689 novel, deleterious variants were identified in the 22 probands at an average of 30 variants per proband (Table 2, Additional files 2: Table S2A, B). Sanger sequencing validated the mapped variants at a validation rate of 83% (53/64), highlighting the reliability of the variants identified by exome mapping analysis (Additional file 1: Table S1D).

**Table 2:** Novel, deleterious variants detected in breast cancer-affected cases*

**Family**	**Total (%)**	**Individual (%)**	Shared**(%)
Family 1			
1	37	35	2
2	26	26	0
3	25	15	10
4	48	39	9
6	29	17	12
7	12	6	6
8	14	6	8
Subtotal	143 (199)	123 (86)	20 (14)
Family 2			
1	22	13	9
2	15	5	10
3	21	9	12
4	21	12	9
5	16	8	8
6	8	2	6
Subtotal	66 (100)	47 (71)	19 (29)
Family 3			
1	39	13	26
2	48	27	21
4	21	12	9
5	32	12	20
6	41	19	22
Subtotal	128 (100)	83 (65)	45 (35)
Total	337 (100)	253 (75)	84 (25)
**Probands**			
1	35	10	25
2	58	22	36
3	74	28	46
4	77	49	28
5	70	28	42
6	41	16	25
7	31	24	7
8	43	27	16
9	51	19	32
10	61	30	31
11	70	35	35
12	51	12	39
13	55	15	40
14	60	30	30
15	51	31	20
16	41	31	10
17	32	18	14
18	57	25	32
19	58	18	40
20	47	23	24
21	33	25	8
22	34	22	12
Total	689 (100)	568 (82)	121 (18)
Per proband	30	26	6

*The counts in subtotal and total are the unique number of variants.

**Shared with family members in the families, or shared with other probands.

### Novel deleterious variants are mostly family-specific

**Figure 2:**
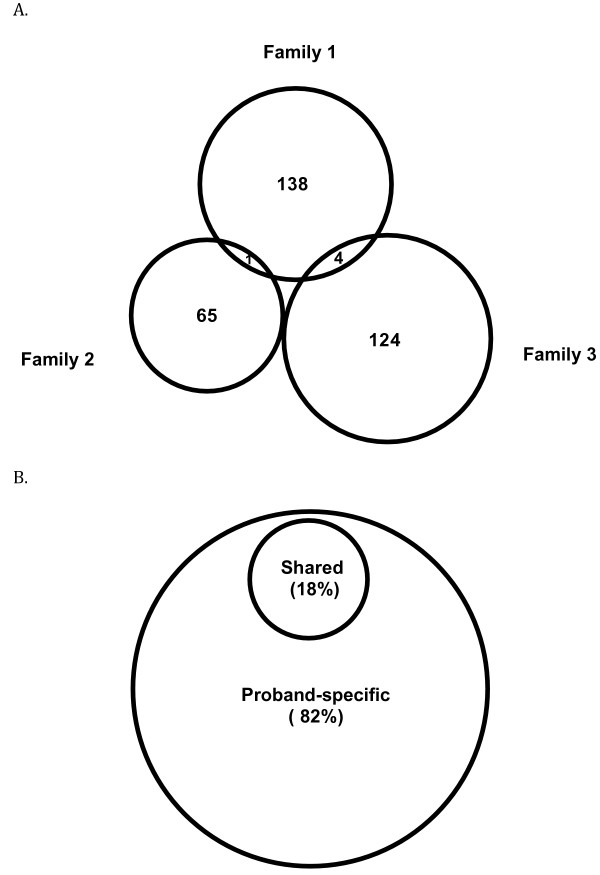
**Comparison of the variants in *BRCAx *families and probands. A**. Comparison in the three families. **B**. Comparison in the probands. The results show that the variants detected in the cancer-affected family members are highly family-specific. The higher rate (18%) of the shared variants in the probands are likely due to the remaining normal variants not filtered in the probands and the larger number of families represented by the probands than the three families.

We compared the variants within each family. We observed that 25% of the variants on average (14% in Family I, 29% in Family II, 35% in Family III) were shared in multiple affected members in each family, whereas 75% on average (86% in Family I, 71% in Family II and 65% in Family III) were present only in single affected member in each family (Table 2). We then compared the shared variants between the three families, and found only 1 variant was shared between Family I and Family II, four variants were shared between Family I and Family III (Figure 2A). For the 689 variants identified in the probands, 82% were proband-specific, and only 18% were shared between probands at various frequencies (Figure 2B, Additional file 2: Table S2A, S2B). The results indicate that the majority of the novel, deleterious variants identified in the three families and probands are family-specific, i.e., present only in each family but not shared with other families.

### Identification of putative genetic predispositions

We analyzed the shared mutations between the affected members of the same family, the functional class of the mutated genes, and existing evidence for their contribution to cancer. In doing so, we identified the variants as the putative predispositions in Family I, II, and III, and probands (Table 3, Additional file 1: Table S1A, S1B, S1C). For Family I, this was the PTEN-Induced Putative Kinase 1 (*PINK1)*; for Family II, these were Lysine (K) Acetyltransferase 6B *(KAT6B)* and Neurogenic Locus Notch Homolog Protein 2 (*NOTCH2)*; and for Family III, this was Phosphorylase Kinase Beta (*PHKB*).

**Table 3:** Putative predispositions in familial breast cancer families and probands

	**Gene**	**Description**	**Position**	**Nucleotide**	**Amino acid**	**Type**	PolyPhen2*		**Cancer-affected member**							**Frequency**
							**Score prediction**									
Family 1									1	2	3	4	6	7	8	
	GPRIN1	G protein regulated inducer of neurite outgrowth 1	chr5:176026123	c.T713C	p.L238S	Exonic	0.91	D	-	+	+	+	+	+	-	5
	PINK1	PTEN induced putative kinase 1	chr1:20972051	c.960-2A > G		Splicing	NA	NA	-	-	+	+	-	-	-	2
	POLK	Polymerase (DNA directed) kappa	chr5:74892737	c.A2219G	p.H740R	Exonic	0.62	P	-	-	-	+	-	-	-	1
Family 2									1	2	3	4	5	6		
	KAT6B	K(lysine) acetyltransferase 6B	chr10:76789128	c.G4546T	p.D1516Y	Exonic	0.95	D	-	+	+	+	+	+		5
	KAT6B	K(lysine) acetyltransferase 6B	chr10:76789311	c.C4729T	p.R1577C	Exonic	0.96	D	-	+	+	+	+	+		5
	NOTCH2	Notch 2	chr1:120459167	c.C6178T	p.R2060C	Exonic	0.99	D	-	-	+	-	-	+		2
Family 3									1	2	4	5	6			
	NANP	N-acetylneuraminic acid phosphatase	chr20:25596725	c.A583G	p.I195V	Exonic	0.98	D	+	-	+	-	-			2
	PHKB	phosphorylase kinase, beta	chr16:47628126	c.1204 + 1G > T		Splicing	NA	NA	-	+	-	+	-			2
Proband																
1	JAKMIP3	Janus kinase and microtubule interacting protein 3	chr10:133955524	c.G1574C	p.G525A	Exonic	1.00	D								
2	POLQ	Polymerase (DNA directed), theta	chr3:121207798	c.A3980C	p.Q1327P	Exonic	1.00	D								
3	DUX2	Double homeobox 2	chr10:135494906			Splicing	NA	NA								
4	UBE2L3	Ubiquitin-conjugating enzyme E2L 3	chr22:21975938	c.G349A	p.E117K	Exonic	0.96	D	.	.						
5	RAD23B	RAD23 homolog B (S. cerevisiae)	chr9:110087260	c.C1028T	p.P343L	Exonic	0.99	D	.	.						
7	GATA3	GATA binding protein 3	chr10:8100630	c.C604T	p.R202C	Exonic	0.92	D								
8	KAT6B	K(lysine) acetyltransferase 6B	chr10:76744854	c.G2390A	p.S797N	Exonic	0.98	D								
9	LIG1	Ligase I, DNA, ATP-dependent	chr19:48637322	c.G1525A	p.E509K	Exonic	0.95	D	.	.						
10	LIG4	Ligase IV, DNA, ATP-dependent	chr13:108862463	c.G1154A	p.R385K	Exonic	1.00	D								
14	NOTCH2	Notch 2	chr1:120529603	c.G854A	p.R285H	Exonic	1.00	D								
15	ABL1	c-abl oncogene 1, non-receptor tyrosine kinase	chr9:133729493	c.G122A	p.G41D	Exonic	0.92	D								
16	TNK2	Tyrosine kinase, non-receptor, 2	chr3:195596385	c.C1760T	p.P587L	Exonic	1.00	D								
17	NFRKB	Nuclear factor related to kappaB binding protein	chr11:129755398	c.G611A	p.R204H	Exonic	1.00	D								
18	NFKBIZ	Nuclear factor of kappa light polypeptide gene enhancer	chr3:101576029			Splicing	NA	NA								
19	SMG1	SMG1 phosphatidylinositol 3-kinase-related kinase	chr16:18879624	c.C3083T	p.T1028M	Exonic	0.99	D								
20	PRKCQ	Protein kinase C, theta	chr10:6528042	c.G855C	p.Q285H	Exonic	1.00	D								
21	ADRA2A	Adrenoceptor alpha 2A	chr10:112838117	c.C363G	p.C121W	Exonic	1.00	D								
22	PPFIA4	Protein tyrosine phosphatase, receptor type	chr1:203025582	c.C668T	p.T223M	Exonic	0.92	D								

D: Probably damaging (score: 0.909-1); P: Possibly damaging (score: 0.447 - 0.908).

*PINK1* is a mitochondrial serine/threonine-protein kinase. Mutation in *PINK1* causes autosomal recessive Parkinson’s disease [22]. *KAT6B* is a histone acetyl transferase involved in DNA replication, gene expression and regulation, and epigenetic modification of chromosomal structure [23]. Mutations in *KAT6B* cause multiple neurological diseases [24]. *NOTCH2* is a member of the Notch family involved in controlling cell fate decision. Low Notch activity leads to hyperproliferative activity in breast cancer [25] and mutation in *NOTCH2* causes Hajdu-Cheney syndrome [26]. PHKB regulates the function of phosphorylase kinase [27]. Mutation in *PHKB* causes glycogen storage disease type 9B [28]. Interestingly, a variant in Polymerase (DNA-Directed) Kappa *(POLK)* was present in Family I member #4. *POLK* is a member of Y family DNA polymerases, and functions by repairing the replication fork passing through DNA lesions [29]. Although we are not able to validate it due to the lack of DNA from the subject’s parents, it raises a possibility that this variant could be a *de novo* mutation in this individual. Multiple transcriptional factors were also affected by the mutations in each family. For example, the following transcriptional factors were mutated in Family I: *ZNF335, LRRC66, ZNF417, ZNF587, GTF2I, ZFAND4, EIF4G2, GZF1, CCDC86, ZSCAN18, ZNF546, TAF1L,* and *LRIG3* (Additional file 1: Table S1A).

The variant data from probands show similar patterns as those of the three families (Table 3). In the 22 probands, four carried variants affecting the genes involved in DNA replication and damaging repair. Those include Polymerase (DNA-directed) Theta (*POLQ)* in Proband #2, RAD23 Homolog B (*S. cerevisiae*) (*RAD23B)* in Proband #3, Ligase I DNA, ATP-dependent (*LIG1*) in Proband #9, and Ligase IV DNA, ATP-dependent *(LIG4)* in Proband #10. POLQ repairs the apurinic sites [30]. RAD23B plays a role in nucleotide excision repair [31]. *LIG1* ligates nascent DNA of the lagging strand, and a mutation in *LIG1* causes replication errors, genome instability, and cancer [32]. *LIG4* catalyzes double-strand break repair by joining non-homologous ends, and mutation in *LIG4* causes *LIG4* syndrome [33]. Several variants are found in well-known oncogenes and tumor suppressor genes, such as GATA Binding Protein 3 (*GATA3)* in Proband #7 and Abelson Murine Leukemia Viral Oncogene Homolog 1 (*ABL1*) in Proband #18. *GATA3* regulates luminal epithelial cell differentiation in the mammary gland [34,35]. The abnormal expression of *GATA3* causes luminal A-type breast cancer [36-38]. ABL1 is a tyrosine kinase that controls cell differentiation and division. It is involved in (9, 22) translocation, forming BCR-ABL fusion gene in chronic myelogenous leukemia (CML) [39]. Several individual variants in different cases affect the same genes but at different positions. For example, in Proband #8, a variant in *KAT6B* (c.G1841A/p.S614N) affects the HAT domain at the N terminal, whereas two variants in *KAT6B* in Family II (c.G3997T/p.D1333Y and c.C4180T/p.R1394C) affect the Met-rich domain at the C-terminal. In Proband #14 and Family II, two different *NOTCH2* variants (c.G854A/p.R285H, c.C6178T/p.R2060C) were present. Multiple variants affect the genes involved in phosphorylation. These include Tyrosine Kinase Non-Receptor 2 (*TNK2*) in proband #16, Phosphatidylinositol 3 Kinase-Related Kinase (*SMG1*) in Proband #19, Protein Kinase C Theta (*PRKCQ*) in Proband #20, and Protein Tyrosine Phosphatase, Receptor Type F *(PPFIZ4)* in Proband #22.

We also performed an analysis at the pathway level by annotating the mutation-affected genes in the three families using KEGG database (http://www.genome.jp/kegg/pathway.html). Certain mutations were identified to affect several functional pathways. For example, the genes mutated in Family I (*ACADVL, AHCY, ALDOA, SGPL1, MAT1A, GALNT8, GGT1*) are involved in metabolic pathways. The genes mutated in Family 2 (*NOTCH2, DUSP16*) are involved in Notch signaling pathway *and* MAPK signaling pathway; genes mutated in Family III (*SLC9A1, ITGAX, ITGAD*) are involved in regulation of actin cytoskeleton.

## Discussion

The majority of families with familial breast cancer lack evidence for their genetic predispositions. Efforts in past decade have made slow progress in determining the unknown genetic predispositions. Currently, population-based approach is adapted as the major promising tool to reach the goal [40]. One weakness of this approach is that it can “dilute out the effects of a very strong association in a small subset of the study population” [41]. It requires a large-size disease population of over tens of thousands but the predispositions identified will likely remain very rare in the disease population. Due to the extreme rarity, such genetic predispositions are often difficult to confirm in different disease populations and to distinguish from normal polymorphisms [5,10]. Our study observed the presence of family-specific, novel, deleterious variants, and putative predispositions in the families and probands analyzed. The information implies that, in addition to the population-based approach, a family-based approach provides another option to determine the genetic predisposition.

**Figure 3:**
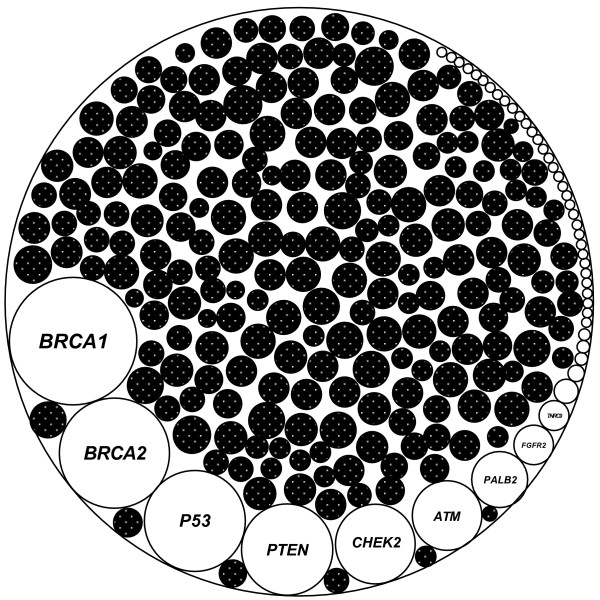
**A model for the genetic predispositions in familial breast cancer.** The known predisposition in *BRCA1* has the highest sharing frequency in the disease population, other known predispositions decrease their frequencies towards extreme rarity in the disease populations, and the family-specific predispositions are enriched in many disease families without known predispositions. The biggest circle represents the entire genetic predispositions in familial breast cancer. The open circles represent the shared, known predispositions, and the black circles represent the family-specific predispositions.

Based on the higher frequencies of well-known predispositions identified by traditional approaches, the rarity of the predispositions recently identified by population-based approach, and the presence of family-specific, novel, deleterious variants in disease families revealed in our study, we propose a model to explain the genetic predispositions in familial breast cancer (Figure 3). In this model, the predisposition in *BRCA1* has the highest frequency in the familial breast cancer population, other known predispositions gradually decrease their frequencies to insignificant levels, and the predispositions for many *BRCAx* familial breast cancers are family-specific. The model explains the difficulty in using traditional and population-based approaches to determine the unknown predispositions, and highlights that applying family-focused approach will be able to determine the genetic predispositions for many *BRCAx* disease families. This model can be further tested in larger number of *BRCAx* familial breast cancer families.

Our study aimed to determine if there are germline mutations present, rather than reach for comprehensive coverage of germline mutations in each family. We achieved this by eliminating all variants matched in population-derived variant databases (i.e., dbSNP137, ESP6500, 1000 genomes) to maximally avoid the variants representing normal polymorphism. Inclusion of such variants as the predisposition candidates, even with the use of certain cut-off such as minor allele frequency (MAF) <0.01, can increase the sensitivity but decrease the specificity of the variants referred to as putative predispositions.

Assignment of a specific mutation as a true predisposition to a disease family requires solid phenotypic evidence from *in vitro* analysis, cell line tests, search of the literature, bioinformatics data analysis, and animal models. This is best evidenced by determining the *BRCA1* germline mutations as genetic predispositions in breast cancer, in which the definitive conclusion for its contribution to breast cancer is based on the mouse models showing development of breast cancer with the germline mutated *BRCA1*[42]. Our current study aims to provide evidence that the *BRCAx* disease families are enriched with germline damaging mutations, such that focusing on each disease family will be required to determine the genetic predisposition in each family. Indeed, even under strict mapping conditions, large numbers of mutations have been detected in each disease family and probands. While the data provide rich resources to identify the true predisposition for the disease family, the data cannot be considered as true predisposition without further phenotypic and functional evidences.

## Conclusions

Our study shows that genetic predispositions in many *BRCAx* familial breast cancer families can be family-specific.

## Abbreviations

BRCAx: Familial breast cancer without known mutations in *BRCA1* and *BRCA2*; Proband: the first affected family member seeking medical attention; Exome sequencing: Sequencing the entire coding region in a genome using the next generation DNA sequencing technology; SAM: Sequence Alignment/Map format used for storing sequence data in a series of tab delimited ASCII columns; BAM: A binary format for storing sequence data in a compressed, indexed, binary form; GATK: Genome Analysis Toolkit. It is a software package to analyse next-generation resequencing data; VarScan 2: a software package to detect variants in next-generation resequencing data; PolyPhen-2: a software to predict possible impact of an amino acid substitution on the structure and function of a protein; Primer3: a software for designing PCR primers; NCBI: The National Center for Biotechnology Information; dbSNP: Single Nucleotide Polymorphism Database; ESP: Exome Sequencing Project; MAF: Minor Allele Frequency.

## Competing interests

The authors declare that they have no competing interests.

## Authors’ contributions

FX, HW, BD performed experiments. YK performed bioinformatics data analysis. CS, DB performed pedigree analysis, identified the study subjects, and prepared DNA samples. JL performed statistical analysis. EAF, SS, KC developed the UNMC Breast Cancer Collaborative Register used in the study [43]. HL and SMW conceived the study. SMW designed the experiment and wrote the paper. All authors read and approved the final manuscript.

## Pre-publication history

The pre-publication history for this paper can be accessed here:

http://www.biomedcentral.com/1471-2407/14/470/prepub

## Supplementary Material

Additional file 1: Table S1 Variants detected in breast cancer-affected members in three *BRCAx* familial breast cancer families. **Table S1A.** Family 1; **Table S1B.** Family 2; **Table S1C.** Family 3; **Table S1D.** Variants shared among the three families; **Table S1E.** Variants validated by Sanger sequencing. Click here for file

Additional file 2: Table S2 Variants identified in 22 probands. **Table S2A.** Variants only in single proband; **Table S2B.** Variants shared among probands. Click here for file
